# Preoperative spirometry and BMI in deep inspiration breath-hold radiotherapy: the early detection of cardiac and lung dose predictors without radiation exposure

**DOI:** 10.1186/s13014-022-02002-9

**Published:** 2022-02-19

**Authors:** Yutaro Koide, Hidetoshi Shimizu, Takahiro Aoyama, Tomoki Kitagawa, Risei Miyauchi, Yui Watanabe, Hiroyuki Tachibana, Takeshi Kodaira

**Affiliations:** 1 Department of Radiation Oncology, Aichi Cancer Center, Kanokoden 1-1, Chikusa-ku, Nagoya, Aichi Japan; 2 Department of Radiation Oncology, Yachiyo Hospital Radiation Therapy Center, Anjo, Japan

**Keywords:** Radiotherapy, Deep inspiration breath-hold, Breast cancer, Spirometry, Pulmonary function test

## Abstract

**Background:**

This study aimed to investigate preoperative spirometry and BMI as early predictors of the mean heart and lung dose (MHD, MLD) in deep inspiration breath-hold (DIBH) radiotherapy.

**Methods:**

Left-sided breast cancer patients underwent breast-conserving surgery followed by DIBH radiotherapy enrolled. Patients who were not available for preoperative spirometry were excluded. One hundred eligible patients were performed free-breathing (FB-) CT and DIBH-CT for plan comparison. We completed the correlative and multivariate analysis to develop the linear regression models for dose prediction. The residuals were calculated to explore the unpreferable subgroups and compare the prediction accuracy.

**Results:**

Among the parameters, vital capacity (VC) and BMI showed the strongest negative correlation with MHD (r = − 0.33) and MLD (r = − 0.34), respectively. They were also significant in multivariate analysis (*P* < 0.001). Elderly and less VC were independent predictors of increasing absolute residuals (AR). The VC model showed no significant difference in AR compared to the model using the CT parameter of lung volume in FB (LV-FB): median AR of the LV-FB model vs. the VC model was 0.12 vs. 0.11 Gy (*P* = 0.79). On the other hand, the median AR of the MLD model was 0.38 Gy, showing no specific subgroups of larger AR.

**Conclusion:**

Preoperative spirometry and BMI are significant predictors of MHD and MLD, respectively. Although elderly and low-VC patients may have larger predictive variations, spirometry might be a substitute for LV-FB as a predictor of MHD.

## Introduction

Whole breast radiotherapy (RT) following breast-conserving surgery (BCS) reduces locoregional recurrence and deaths from breast cancer [1]. On the other hand, left-sided breast cancer patients have suffered cardiac toxicity depending on the irradiation dose [2–7]. Darby et al. showed that the mean heart dose (MHD) was correlated to the frequency of major coronary events at a rate of 7.4% per Gy with no apparent threshold [6]. Deep inspiration breath-hold (DIBH) is an effective method for reducing cardiac dose compared to free-breathing (FB-) RT [8–13]. Several studies have attempted to identify suitable patients or predict MHD in DIBH using anatomical information based on the simulation CT (e.g., maximum cardiac distance, cardiac contact distance) [14–26]. However, some non-CT parameters such as BMI or pulmonary function test results (PFT) are also reported to correlate to MHD [14, 27–32]. Such non-CT parameters may have the advantage of being available earlier without additional burden or radiation exposure to the patient. Hjelstuen et al. demonstrated that PFT could be used to identify the suitable patients for DIBH instead of using the lung volume (LV) on FB-CT (LV-FB): they estimated LV by using multiple PFTs (spirometry, diffusion capacity for carbon monoxide, and whole-body plethysmography), and confirmed a strong correlation of LV-FB with MHD, and the estimated LV with LV-FB. Although the interval between PFTs and simulation CT was shortly 28.5 h in median, it was an important finding that non-CT parameters could be substituted for the CT parameter to select the suitable patients in DIBH [27]. We hypothesized that even preoperative PFTs could be used instead of LV-FB to develop the earlier prediction.

A few studies have examined the relationship between DIBH and the lung dose, which reported that DIBH seemed to have some advantages in decreasing the lung dose [14, 33]. One study achieved to predict mean lung dose (MLD) using the synthetic DIBH-CT generated with a deep learning approach [34]. Lung dose in breast cancer patients has been associated with an increase in radiation-related lung cancer. In a meta-analysis of breast cancer radiotherapy, the risk of radiation-related lung cancer increased by about 11 percent per Gy MLD [7]. Pneumonitis has also been reported to be related to lung dose, and it is essential to keep lung dose as low as possible in breast cancer radiotherapy [35, 36]. Still, unfortunately, no previous studies found promising non-CT parameters related to the lung dose.

The purpose of this study is to find non-CT predictors in preoperative information (BMI, PFT) associated with the cardiac and lung dose advantages in DIBH. We also aimed to develop the linear regression models to predict MHD and MLD and compared the prediction accuracy with the LV-FB model.

## Materials and methods

### Patient selection and treatment

Our institutional review board approved this study. All participants provided written informed consent and met the following eligibility criteria: histologically proven diagnosis of invasive ductal carcinoma or carcinoma in situ of the left breast, underwent breast-conserving surgery followed by DIBH-RT from June 2018 to September 2021. Patients who do not have available preoperative PFT data were excluded.

### Preoperative pulmonary function test

**Fig. 1:**
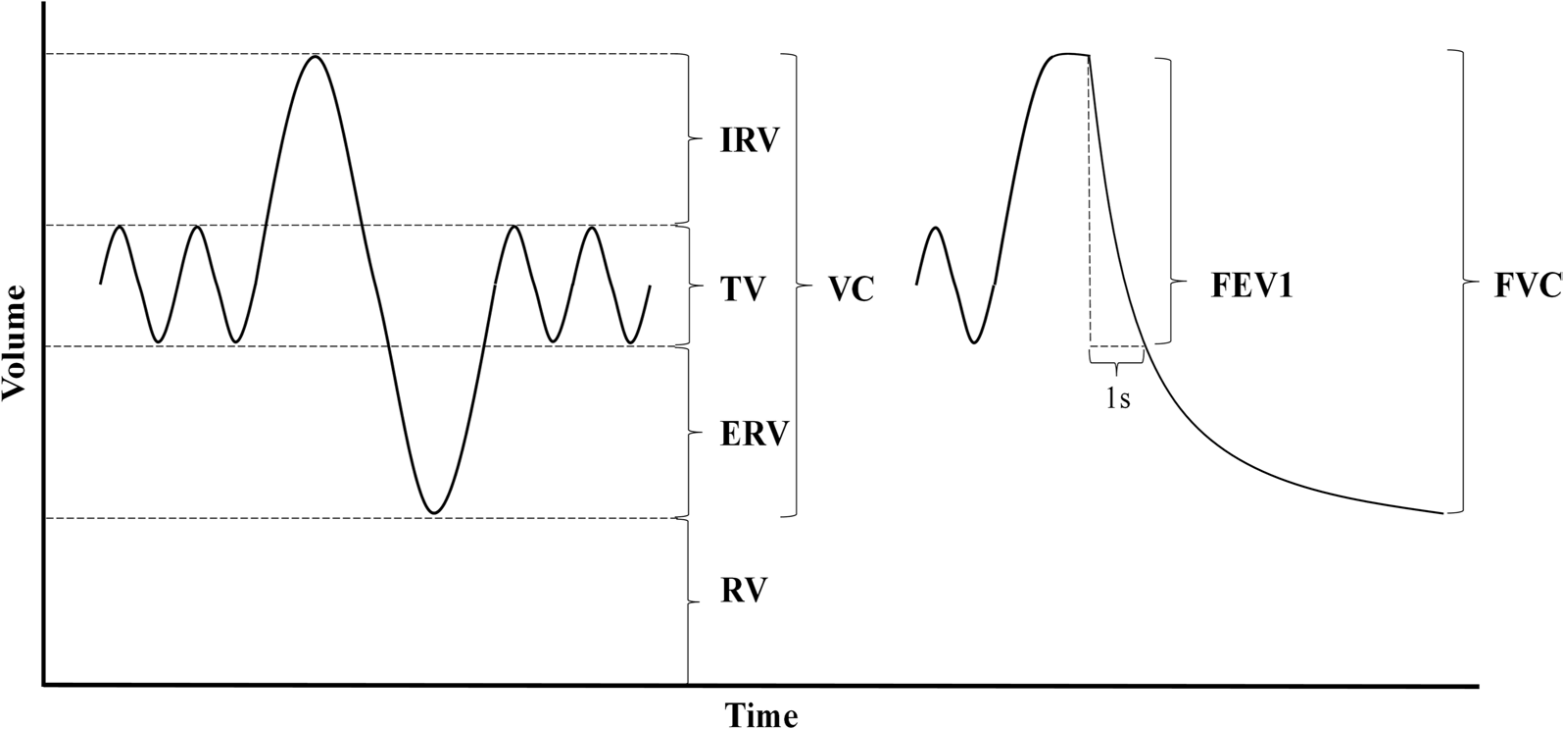
The measurements description of spirometry. IRV: inspiratory reserve volume, TV tidal volume, ERV: expiratory reserve volume, VC: vital capacity, RV: reserve volume, FEV1: forced expiratory volume in 1 s, FVC: forced vital capacity

We perform spirometry as preoperative PFT. Spirometry measures the lung capacities and flow speed of breathing [39]. Figure 1 shows the parameters of the lung capacities: Vital capacity (VC), Inspiratory reserve volume (IRV), Tidal volume (TV), Expiratory reserve volume (ERV). Residual volume (RV) is the volume of air remaining in the lungs after a maximal exhalation, which cannot be directly measured by spirometry. The following parameters of flow speed are also obtained in this study: forced vital capacity (FVC), forced expiratory volume in 1s (FEV1), and peak expiratory flow (PEF), which means the highest forced expiratory flow. The preoperative height and weight are measured at the same of spirometry.

### Planning CT simulation

We performed the same DIBH-RT method on all patients by implementing a technique proposed by Bartlett et al. [11]. Patients were trained to inhale, exhale, and hold deep breaths. The breath-hold training was initially for 5–10 s, and then the breath-hold time was increased up to 20 s. The simulation and training time took about 20–30 min per patient. After confirming the respiratory motion, we marked the points along the midline of the chest with black ink, and the extent to which the body moved from the FB position was ascertained. Then, all patients underwent planning CT scans (FB-CT, DIBH-CT) in the supine position on a wing board with their arms stretched overhead with an Aquilion LB CT system (Canon Medical Systems, Tochigi, Japan). The CT slice thickness was 3 mm.

### Treatment planning on FB-CT and DIBH-CT

**Fig. 2:**
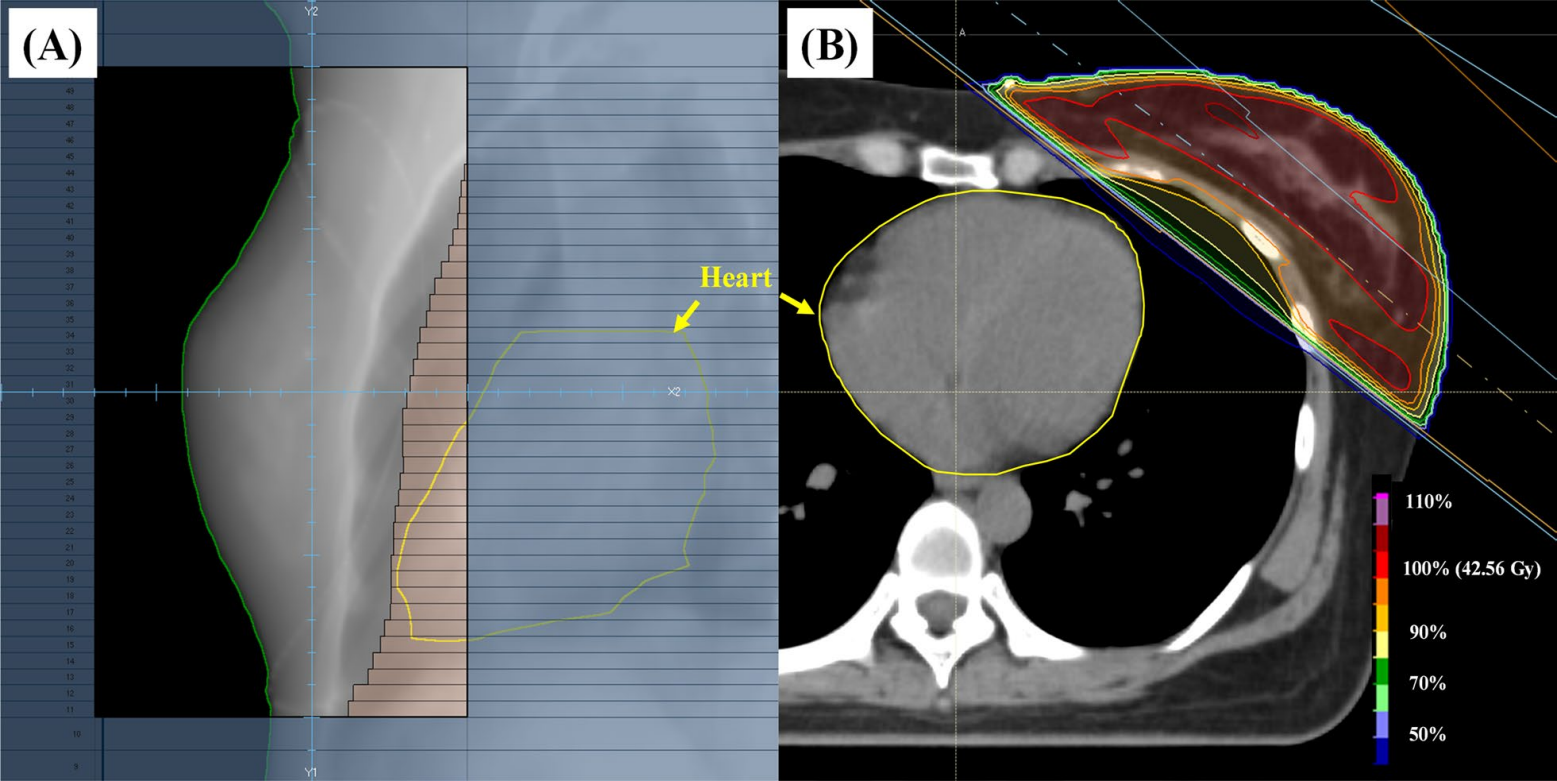
An example of treatment planning. **A** A digitally reconstructed graph (DRR) and **B** A dose distribution map of a typically case of this study. This case was treated in two opposing tangential beams (gantry angle: 128 and 305 degree, collimator angle: 0) with field-in-field technique. The 5.0 mm leaf width multileaf collimator was used

We perform the contouring and dose planning on FB- and DIBH-CT using RayStation version 9 (RaySearch Laboratories AB, Stockholm, Sweden) with the calculation algorithm of Collapsed Cone version 5.1. The prescription dose for the planning target volume (PTV) was 42.56 Gy in 16 fractions using the Varian TrueBeam system (Varian Medical Systems, Palo Alto, USA). We defined clinical target volume (CTV) on each CT, referring to the consensus guidelines [37]. The PTV included the CTV and a 5-mm margin in all directions. The lung contours were automatically created using a model-based segmentation (MBS) function in RayStation. The heart was contoured according to the heart atlas validation study [38]. Three-dimensional conformal RT comprised the treatment planning. All plans consisted of two opposing tangential beams and two additional beams using the field-in-field technique. The two treatment plans were restricted to obtain the beam angle connecting the midline of the patient's chest to the mid-axillary line, and the multileaf collimator (MLC) margin was unified. Figure 2 shows a digitally reconstructed radiograph (DRR) and a dose distribution map of a typical case.

### Statistical analyses

We defined the mean heart dose (MHD) and the mean lung dose (MLD) as the primary objective variables. The explanatory variables are preoperative and postoperative BMI (pre_BMI, post_BMI) and PFT results (VC, TV, IRV, ERV, FVC, FEV1, PEF). The variance inflation factor (VIF) of each two explanatory variables was calculated to verify the multicollinearity. We remove some variables not to exceed the maximum acceptable level of VIF (= 5).

Statistical analysis was performed using R version 3.6.1 (The R Foundation for Statistical Computing, Vienna, Austria). The correlation between the covariates and the objective variables (MHD or MLD) was tested using Pearson's s correlation coefficient; r. The required sample size was calculated as follows: Past studies showed r = − 0.25 to − 0.81 between covariates and MHD [14, 27–32], and if this study shows the minimum r = − 0.25, ninety-seven patients were required with alpha = 0.05 and power = 0.80. The significantly correlated factors with MHD or MLD will be examined using multivariable analyses. A linear regression model was created using the variable with the highest absolute value of the correlation coefficient. Residuals of each regression model were calculated to evaluate and compare the prediction performance. We also looked for a specific patient group with high variability from the scatter plot and compared the absolute errors between patient groups using the Mann–Whitney *U* test. *P* < 0.05 was considered statistically significant.

## Results

### Dataset

In one hundred and thirteen patients enrolled in the study, 100 patients were eligible with available PFT results. Table 1 shows the characteristics of the eligible patients. The median age was 52 years (range: 30 to 76 years, 25% were > 60 years), and the median pre_BMI was 21.6 (range: 16.7 to 39.1). All patients underwent BCS followed by DIBH-RT. Spirometry was performed before BCS, and the interval between spirometry and a simulation CT was a median of 85 days (range: 25 to 258 days). Fifteen patients received preoperative chemotherapy, all of whom underwent spirometry after chemotherapy but before surgery. Ten patients who received postoperative chemotherapy had significantly longer intervals between the spirometry and the simulation CT (mean: 85 vs. 189 days, *P* < 0.001).

**Table 1:** Patient characteristics

Characteristic	Eligible patients (N = 100)
Age: median (range), y > 60 vs. ≤ 60	52 (30–76) 25 vs. 75
Preoperative BMI: median (range), kg/m^2^	21.6 (16.7–39.1)
Postoperative BMI: median (range), kg/m^2^	21.3 (16.7–42.9)
The interval between spirometry and CT, median (range), days	85 (25–258)
Tumor site	
Inner-upper (A)	19
Inner-lower (B)	8
Outer-upper (C)	58
Outer-lower (D)	13
Center (E)	2
TNM:	
Tis	14
T1N0	63
T2N0	12
T1-2N1	10
Other	1
Molecular subtypes:	
	70
HER2 (HR negative and HER2 positive) Luminal (HR-positive and HER2 negative)	9
Luminal HER2 (HR and HER2 positive)	9
Triple-negative (HR and HER2 negative)	9
Unknown or other	3
Neoadjuvant chemotherapy, Y/N	15/85
Surgery:	
BCS alone	6
BCS + SLNB (No ALND)	89
BCS + ALND	5
Adjuvant chemotherapy, Y/N	10/90
Spirometry: median (range), ml	
VC	3.01 (1.94–3.9)
TV	0.71 (0.13–1.68)
IRV	1.30 (0.40–2.04)
ERV	0.95 (0.03–1.74)
FVC	3.00 (1.90–3.90)
FEV1	2.35 (1.42–3.35)
PEF	5.61 (2.60–8.62)
Total lung capacity on CT: median (range), ml	
FB	2315 (827–3856)
DIBH	3701 (1975–5328)

BMI: body mass index, BCS: breast-conserving surgery, SLNB: sentinel lymph node biopsy, ALND: axillary lymph node dissection, VC: vital capacity, TV tidal volume, IRV: inspiratory reserve volume, ERV: expiratory reserve volume, FVC: forced vital capacity, FEV1: forced expiratory volume in 1 s, PEF peak expiratory flow, FB: free-breathing, DIBH: deep inspiration breath-hold

Since the pairs of pre_BMI vs. post_BMI (VIF = 125), FEV1 vs. FVC (VIF = 6.02), and VC vs. FEV1 (VIF = 5.95) exceeded the maximum acceptance VIF level, post_BMI, FVC, and FEV1 were excluded from the following statistical analysis. The remaining explanatory variables are Age, pre_BMI, VC, TV, IRV, ERV, and PEF.

### Statistical results for the mean heart dose

**Fig. 3:**
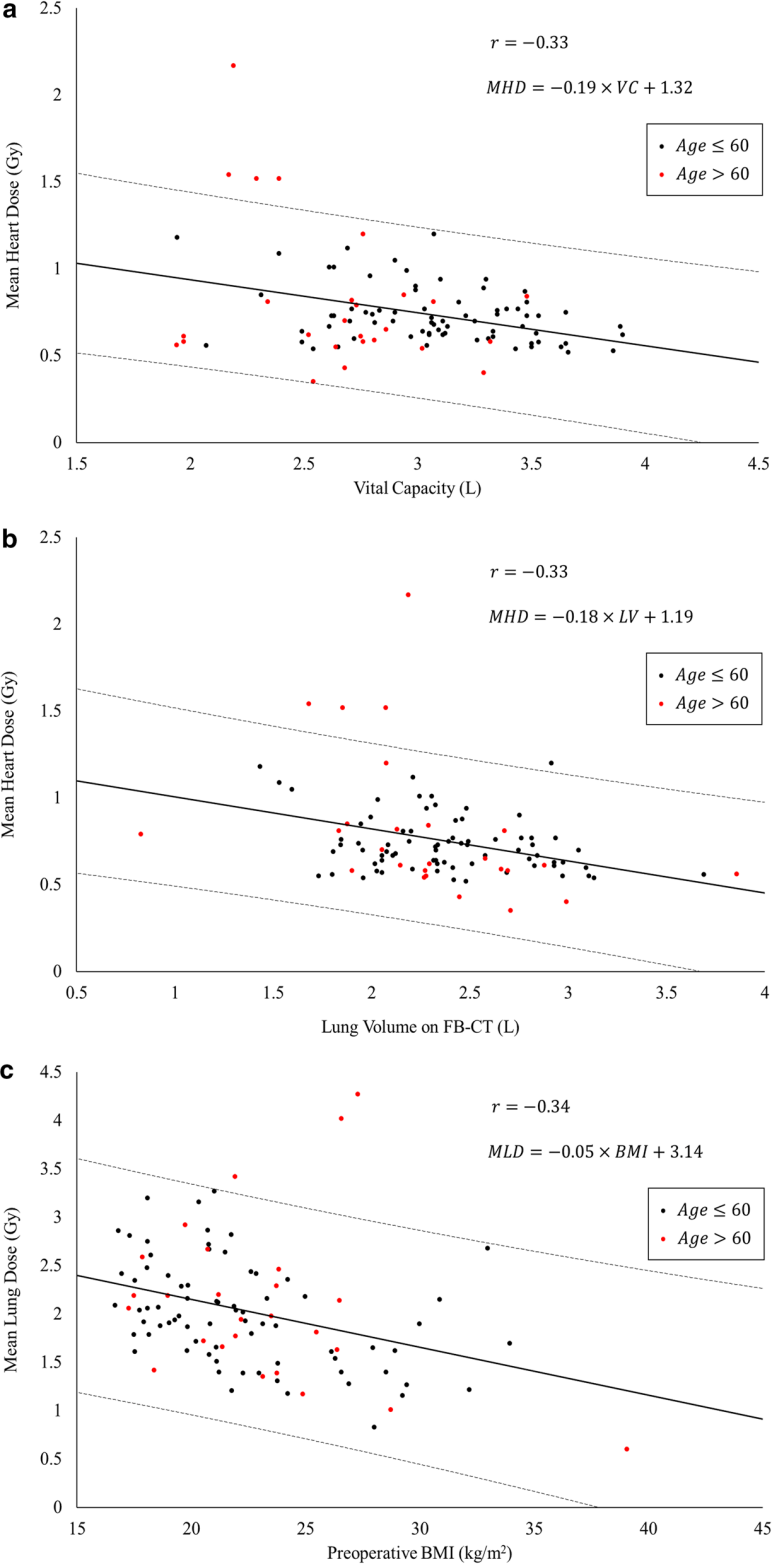
The scatter plot of variables and a regression line. **a** The mean heart dose (MHD) and the vital capacity in spirometry (VC), **b** MHD and the lung volume on free-breathing CT (LV), **c** The mean lung dose (MLD) and preoperative BMI. r: correlation coefficient

The results of the correlation analysis with MHD and MLD as the objective variables are shown in Table 2. Among the remaining explanatory variables, VC showed the strongest correlation with MHD (r = − 0.33, *P* < 0.001, Fig. 3a) and was also an independent and significant predictor in multivariate analysis (*P* < 0.001). All other variables were not statistically significant. The following specifies the linear regression fitting model of MHD.

**Table 2:** The correlation analysis between the doses and preoperative parameters

Characteristic	Mean heart dose r, *P*	Mean lung dose r, *P*
Age	0.0807, *P* = 0.43	− 0.0616, *P* = 0.54
Pre_BMI	0.0162, *P* = 0.87	− 0.336, *P* < 0.001*
VC	− 0.330, *P* < 0.001*	− 0.0395, *P* = 0.70
TV	− 0.145, *P* = 0.15	0.0397, *P* = 0.70
IRV	− 0.168, *P* = 0.095	− 0.209, *P* = 0.037*
ERV	− 0.103, *P* = 0.31	0.141, *P* = 0.16
PEF	− 0.106, *P* = 0.29	− 0.110, *P* = 0.28

Pre_BMI: preoperative body mass index, VC: vital capacity, TV tidal volume, IRV: inspiratory reserve volume, ERV: expiratory reserve volume, PEF peak expiratory flow, r: Pearson's correlation coefficient, * means statistically significant

$$MHD \left(Gy\right)=-0.190\times VC (L)+1.317$$

The median (range) absolute residuals were 0.10 (0.0090–1.27) Gy: older age and less VC were independent predictors with increasing the absolute residuals (*P* < 0.001). When differentiated by age, patients older than 60 years had significantly greater variability: Median value (range) of the residuals in patients > 60 vs. < 60 years were 0.21 (0.0090–1.27) Gy vs. 0.10 (0.00090–0.46) Gy (*P* < 0.001).

LV-FB was significantly correlated to MHD (r = − 0.33, *P* < 0.001, Fig. 3b). However, the following LV-FB linear regression model did not show significantly different residuals compared to the VC model: median values (range) of the absolute residuals were 0.12 (0.0023–1.37) Gy vs. 0.11 (0.00090–1.27) Gy (LV-FB vs. VC, *P* = 0.79).

$$MHD \left(Gy\right)=-0.180\times LVFB (L)+1.190$$

### Statistical results for the mean lung dose

On the other hand, pre_BMI showed the strongest correlation with MLD (r = − 0.34, *P* < 0.001, Fig. 3c) and was also a significant independent predictor in multivariate analysis (*P* < 0.001). All other variables were not statistically significant. The linear regression model for MLD is specified below.

$$MLD (Gy)=-0.050\times pre\_BMI (kg/{m}^{2})+3.141$$

The median (range) absolute residuals of the pre_BMI model was 0.38 (0.0023–2.49) Gy. In contrast to MHD, there was no specific groups with larger residuals: the variability between the elderly and non-elderly in MLD: Median value (range) of the absolute residuals in patients > 60 vs. < 60 years were 0.39 (0.0023–2.49) Gy vs. 0.37 (0.0036–1.19) Gy.

## Discussion

In this study, we investigated the relationship of non-CT parameters to MHD and MLD in DIBH. Although BMI and PFT have also been used as exploring parameters in previous studies [14, 27–32], this study used only preoperative information for the first time. Thus, the prediction results can be extrapolated earlier, which may help determine the initial surgical treatment or postoperative radiotherapy. Also, the parameters of this study were obtained as a routine workup, so additional patient burden or radiation exposure would not occur. Compared to the previous studies that used non-CT parameters as described in Table 3 [14, 27–32], this study has some strong points: a large sample size, investigating the relationship to MLD as well as MHD.

**Table 3:** Reference of past similar studies

Authors	Year	Sample size	Objective variable	Exploring variable		Pearson's correlation coefficient
Hjelstuen [27]	2015	16, 18 (two cohorts)	MHD LV-FB		LV-FB PFT	− 0.81
						0.85
Lee [28]	2016	88	MHD		LV-FB	− 0.59
Cao [14]	2019	67	**Δ**MHD MLD		**Δ**LV LV-FB	0.44
						0.33
Yamauchi [29]	2020	85	**Δ**MHD		BMI	0.25
Mkanna [30]	2018	103	**Δ**MHD		BMI	0.32
Czeremszynska [31]	2017	31	**Δ**MHD **Δ**MHD		BMI LV-FB	0.44
						− 0.58
Browne [32]	2020	31	MHD		BMI	0.51

MHD: mean heart dose, **Δ**MHD: mean heart dose difference between free-breathing and deep inspiration breath-hold, LV: the lungs volume, LV-FB: the lungs volume on free-breathing CT, **Δ**LV: LV difference between deep inspiration breath-hold and free-breathing, PFT: pulmonary function test, MLD: mean lung dose, BMI: body mass index, r: correlation coefficient

VC was negatively correlated with MHD (r = − 0.34), the strongest correlation among the preoperative factors; this correlation value was unexpectedly low since Hjelstuen et al. showed a strong correlation of 0.8 or higher [27]. One of the possible reasons for this gap could be the small sample size (16–18 patients) in their study. Small sample studies may have a significant sampling error, so appropriate sample sizes should be prepared based on calculations: if ten times of random subsampling of 16 patients, our cohort showed r in the range of –0.77 to 0.44 (median: –0.31). Another possible reason is that our cohort has different patient characteristics from theirs. A systematic review shows that the cardiac dose from DIBH has tended to be lower in recent years [40], with a maximum cardiac dose of about 4 Gy in this study and 10–14 Gy in theirs. Furthermore, we found that older age and lower VC were associated with larger residuals in the predicted results, so the inclusion of many elderly patients may be one reason why the correlation values were lower than expected. If patients aged > 60 were excluded, the correlation values slightly improved (r = − 0.36). Spirometry cannot measure RV, so the difference between VC and LV-FB might be more significant in elderly or low VC patients expected to have a large RV.

Previous studies showed the correlation between LV-FB and MHD [14, 27, 28], and Hjelstuen et al. confirmed that PFT could be used as a surrogate for LV-FB to select suitable patients of DIBH. However, their PFT must be performed additionally according to the planned CT schedule, and multiple types of PFT are necessary. Furthermore, compared to their study, the interval between CT and PFT in our study was significantly different, with a median of 28.5 h (range: 0.5–528 h) vs. 85 days (25–258 days). Our study showed that preoperative spirometry alone is also a substitute for LV-FB as a predictor of MHD.

Although three studies have reported a negative correlation between BMI and MHD [29–31], this study did not show a significant correlation between them. On the other hand, BMI negatively correlated with MLD (r = − 0.33). To investigate the reason for the negative correlation, we focused on the LV change from FB to DIBH, which correlated with BMI (r = 0.31, *P* < 0.001). Thus, the lungs of high BMI patients expand greater than those of low-BMI patients, possibly resulting in the higher dose sparing benefit.

There are several limitations to this study. First, the patients in this study were mainly those who underwent BCS with good pulmonary function. All patients were performed DIBH-CT simulations regardless of their pulmonary function or BMI results. Therefore, it is still unclear whether our results can be extrapolated to patients undergoing chest wall or lymph node irradiation. Second, although the correlation coefficients obtained in this study showed statistical significance with an appropriate sample size, the correlation value is not strong, so further exploration of other preoperative parameters would be desirable for more accurate prediction. Third, there is a large variability in the interval between the preoperative PFT examination and the simulation CT.

In conclusion, preoperative spirometry and BMI are significant predictors of MHD and MLD, respectively. Although the elderly and low-VC patients may have larger predictive variations, spirometry might be a substitute for LV-FB as a predictor of MHD.

## Data Availability

Research data are stored in an institutional repository and anonymized numerical data will be shared upon request to the corresponding author. Research image data are not available at this time.

## Abbreviations

BMI: Body mass index
MHD: Mean heart dose
MLD: Mean lung dose
DIBH: Deep inspiration breath-hold
CT: Computed tomography
AR: Absolute residuals
FB: Free-breathing
RT: Radiotherapy
BCS: Breast-conserving surgery
PFT: Pulmonary function test
LV: Lung volume
VC: Vital capacity
IRV: Inspiratory reserve volume
TV: Tidal volume
ERV: Expiratory reserve volume
RV: Residual volume
FVC: Forced vital capacity
FEV1: Forced expiratory volume in 1 s
PEF: Peak expiratory flow
PTV: Planning target volume
CTV: Clinical target volume
MBS: Model-based segmentation
MLC: Multileaf collimator
DRR: Digitally reconstructed radiograph
VIF: Variance inflation factor
